# Disruption of outer blood-retinal barrier by *Toxoplasma gondii*-infected monocytes is mediated by paracrinely activated FAK signaling

**DOI:** 10.1371/journal.pone.0175159

**Published:** 2017-04-13

**Authors:** Hyun Beom Song, Hyoung-Oh Jun, Jin Hyoung Kim, Young-Ha Lee, Min-Ho Choi, Jeong Hun Kim

**Affiliations:** 1 Fight against Angiogenesis-Related Blindness (FARB) Laboratory, Biomedical Research Institute, Seoul National University Hospital, Seoul, Republic of Korea; 2 Department of Biomedical Sciences, College of Medicine, Seoul National University, Seoul, Republic of Korea; 3 Department of Parasitology and Tropical Medicine, Seoul National University College of Medicine, and Institute of Endemic Diseases, Seoul National University Medical Research Center, Seoul, Republic of Korea; 4 Department of Infection Biology, Chungnam National University School of Medicine, Daejeon, Republic of Korea; 5 Department of Ophthalmology, College of Medicine, Seoul National University, Seoul, Republic of Korea; University at Buffalo, UNITED STATES

## Abstract

Ocular toxoplasmosis is mediated by monocytes infected with *Toxoplasma gondii* that are disseminated to target organs. Although infected monocytes can easily access to outer blood-retinal barrier due to leaky choroidal vasculatures, not much is known about the effect of *T*. *gondii-*infected monocytes on outer blood-retinal barrier. We prepared human monocytes, THP-1, infected with *T*. *gondii* and human retinal pigment epithelial cells, ARPE-19, grown on transwells as an *in vitro* model of outer blood-retinal barrier. Exposure to infected monocytes resulted in disruption of tight junction protein, ZO-1, and decrease in transepithelial electrical resistance of retinal pigment epithelium. Supernatants alone separated from infected monocytes also decreased transepithelial electrical resistance and disrupted tight junction protein. Further investigation revealed that the supernatants could activate focal adhesion kinase (FAK) signaling in retinal pigment epithelium and the disruption was attenuated by FAK inhibitor. The disrupted barrier was partly restored by blocking CXCL8, a FAK activating factor secreted by infected monocytes. In this study, we demonstrated that monocytes infected with *T*. *gondii* can disrupt outer blood-retinal barrier, which is mediated by paracrinely activated FAK signaling. FAK signaling can be a target of therapeutic approach to prevent negative influence of infected monocytes on outer blood-retinal barrier.

## Introduction

Blood-retinal barrier (BRB) is composed of tight junctions of retinal capillary endothelial cells (inner BRB) and retinal pigment epithelial cells (outer BRB). The retinal pigment epithelium (RPE) is a monolayer of highly specialized hexagonal-shaped cells located between the sensory retina and the leaky choroidal vasculature. The tight junctions between RPE cells efficiently restrict paracellular permeation into the retina [1]. A disruption can increase permeability and impair homeostatic function of RPE leading to visual disturbance.

One of the conditions where the barriers are impaired is uveitis. According to the experimental models of uveitis, leukocyte infiltration and BRB breakdown primarily occurred at inner BRB while packed inflammatory cells at choroid without evidence of migration were observed at outer BRB [2]. At later stages of uveitis, occasional infiltration of inflammatory cells and opening of tight junctions were observed at outer BRB [2, 3]. As ocular toxoplasmosis is the most common cause of posterior uveitis [4], similar impairment is expected in ocular toxoplasmosis.

Ocular toxoplasmosis is caused by infection with *Toxoplasma gondii*, an obligate intracellular protozoan parasite. Following the ingestion of cysts consisting of *T*. *gondii*, tachyzoites, invasive and fast replicative forms of *T*. *gondii*, cross the intestinal epithelium and infect monocytic cells adjacent to the lamina propria [5]. The infected cells are disseminated through the blood flow toward target organs. Acute ocular toxoplasmosis commonly appears as necrotising retinochoroiditis that shows diffuse inflammation in the retinal and RPE/choroidal tissue [6], and there are various other presentations such as punctate outer retinal toxoplasmosis whose lesion is restricted to RPE. According to the experimental study of parasite load in various parts of the eye after oral administrations, *T*. *gondii* was simultaneously detected in the retina and choroid [7]. The load in the choroid was remaining at low level, while the load in the retina increased gradually. These findings contradict conventional concept of primary involvement of retina and secondary involvement of choroid by *T*. *gondii* infection [8].

It is interesting to note that parasite load in the choroid remained at low level [7]. The gradual increase of the parasite load in the retina probably indicate transmigration and replication of *T*. *gondii*. As it is known that *T*. *gondii* inside RPE cells can replicate [9], low level of parasite load in the RPE/choroid probably indicate migration of infected cells to the RPE/choroid can occur but replication in the RPE/choroid is likely to be restricted. Therefore, pathogenesis of lesions at outer BRB was investigated by using *T*. *gondii*-infected cells instead of tachyzoites that were commonly used in other studies to directly infect RPE [10–12].

In this study, we demonstrated disruption of an *in vitro* model of outer BRB by infected monocytes. Supernatants alone could also disrupt outer BRB and the disruption was attenuated by focal adhesion kinase (FAK) inhibitor. CXCL8 secreted from infected monocytes partly mediated the disruption of outer BRB.

## Materials and methods

### Cell culture

Human RPE cells, ARPE-19, were purchased from American Type Culture Collection (Manassas, VA, USA) and maintained in Dulbecco's modified Eagle's medium: nutrient mixture F-12 (DMEM/F12) containing 10% fetal bovine serum (FBS), 100 U/mL penicillin, and 100 μg/mL streptomycin. Human monocytic cells, THP-1, were purchased from Korean Cell Line Bank (Seoul, Korea) and maintained in RMPI 1640 containing 10% FBS, 100 U/mL penicillin, and 100 μg/mL streptomycin. Human foreskin fibroblasts (HFF) were purchased from Korean Cell Line Bank and maintained in Dulbecco's modified essential medium containing 10% FBS, 100 U/mL penicillin, and 100 μg/mL streptomycin. All culture reagents were purchased from Invitrogen (Carlsbad, CA, USA). Cells were incubated at 37°C in a moist atmosphere of 95% air and 5% CO_2_.

### Parasites and reagents

An avirulent type 2 strain of *T*. *gondii* expressing green fluorescent protein (PTG strain, ATCC #50941) [13] was purchased from the American Type Culture Collection. Tachyzoites were maintained by serial passaging in HFF.

FAK was inhibited by treating 1 μM of PF-573228 (Tocris, Minneapolis, MN, USA) on apical side of RPE monolayer and CXCL8 was neutralized by treating 1 μg/mL of human CXCL8 antibody (MAB208; R&D systems, Minneapolis, MN, USA) on basolateral side of RPE monolayer. An antibody with corresponding IgG_1_ isotype (MAB002; R&D systems) was used as a control.

### Infection of cells with *Toxoplasma gondii*

Infected HFFs were lysed and filtered through a 3-μm pore-sized membrane to remove host cell debris. Prepared tachyzoites were added to THP-1 cells at a density of 10^6^ cells/mL (parasite/cell ratio 2). After incubation for 24 h at 37°C in an atmosphere containing 5% CO_2_, infection was confirmed under the fluorescence microscope. Then, supernatants were separated by centrifugation at 1000g for 10 min and filtration through a 0.22-μm pore-sized filter. The collected supernatants were stored at -20°C. Heat-inactivated tachyzoites were prepared after incubation at 56°C for 30 min as previously described [14, 15]. For immunocytochemistry, THP-1 cells were labeled with CellTracker^™^ Deep Red (Invitrogen) prior to the addition of the tachyzoites.

### Transepithelial electrical resistance measurement

RPE cells were plated at a density of 1.6×10^5^ cells/cm^2^ on transwell filters (6.5-mm diameter, 0.4-μm pore, Corning Inc., NY, USA) coated with laminin. After confluency was achieved, the monolayer was maintained in DMEM/F12 containing 1% FBS to induce polarization [16]. Monolayers incubated more than 4 weeks were utilized for the analysis of barrier function and immunocytochemical staining.

The transepithelial electrical resistance (TEER) was measured with an eipithelial voltohmmeter EVOM2 (World Precision Instruments, Sarasota, FL, USA). The value of each individual transwell was calculated by subtracting the value of a coated transwell without cells. Values measured immediately after treatments were set as 100% to normalize the results.

### Immunocytochemistry

After removal of the medium, the monolayers were washed with PBS and fixed with 4% paraformaldehyde at room temperature for 15 min. After permeabilization with 0.2% Triton X-100 in PBS at room temperature for 10 min, cells were incubated with 1% bovine serum albumin in PBS at room temperature for 1 h. Cells were then incubated overnight at 4°C with rabbit anti-ZO-1 (Invitrogen). On the next day, cells were washed with PBS and incubated at room temperature for 1 h with secondary antibodies (Alexa Fluor 488 anti-rabbit IgG, Invitrogen). The filters were detached from the transwells, mounted on slide glasses and observed under fluorescence microscope (Ni-U, Nikon, Tokyo, Japan)

### Western blot analysis

Western blot analysis was performed using standard methods. After cell lysates were prepared in RIPA buffer, equal amounts of protein were separated by electrophoresis on 7% sodium dodecyl sulfate—polyacrylamide gel electrophoresis and transferred to nitrocellulose membrane (Amersham, Pittsburgh, PA, USA) by electrotransfer. After being blocked with skim milk or bovine serum albumin, the membranes were incubated overnight at 4°C with rabbit anti-phospho-FAK(Y^397^), anti-FAK, anti-β-actin (all three from Cell Signaling Technology, Beverly, MA, USA), rabbit anti-ZO-1 or anti-occludin (both from Invitrogen). After incubation with HRP-linked anti-rabbit IgG secondary antibody, the immunoreactive bands were visualized using a chemiluminescent kit solution (Dogen, Seoul, Korea).

### Quantification of cytokines secreted by THP-1 cells in response to *Toxoplasma gondii* infection

Supernatants from infected THP-1 were collected as described above. Levels of CXCL8 were measured by using ELISA kits (KOMA Biotech, Seoul, Korea) according to manufacturer’s instructions. The minimum detectable concentration was 16 pg/mL according to manufacturer’s descriptions. In addition, supernatants were analyzed by Human Cytokine Array kit (ARY005B; R&D systems) according to manufacturer’s instructions.

### Statistics

Differences between groups were evaluated with the Mann-Whitney U-test using SPSS 19.0 (SPSS Inc., Chicago, Illinois, USA). Mean values with SEM were described. P-values less than 0.05 were considered to be statistically significant.

## Results and discussion

### Infected monocytes disrupt tight junction proteins

A previous study revealed that tachyzoites probably take a paracellular pathway to cross intestinal epithelial barriers leaving the integrity of barriers unaltered [17]. Outer BRB is also constituted by epithelial cells, but the results from intestinal barriers cannot be applied to outer BRB because parasite dissemination is mainly fulfilled not by extracellular tachyzoites but by intracellular tachyzoites in leukocytes [18]. Another study revealed CD11b^+^ monocytes are responsible for carrying tachyzoites in the blood and transporting them to the brain [19]. Therefore, we utilized monocytes infected with tachyzoites to investigate their effects on integrity of our *in vitro* model of outer BRB.

**Fig 1:**
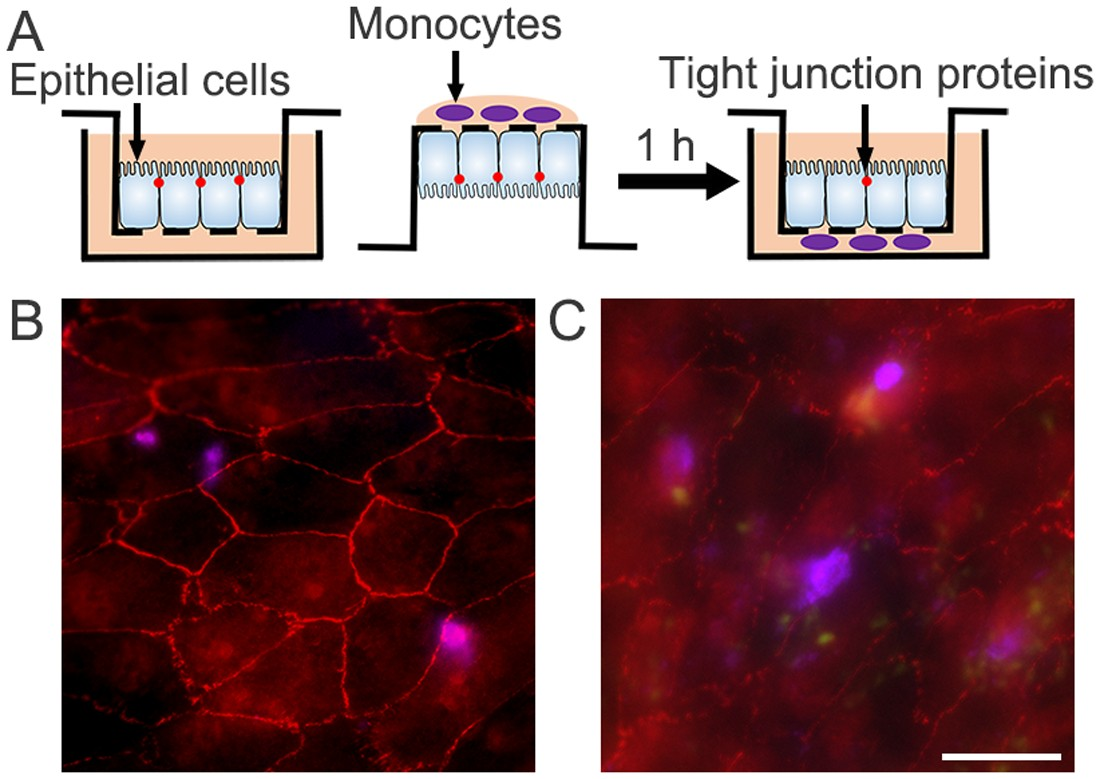
Infected monocytes disrupt tight junction protein. Human monocytic cells, THP-1, were labeled with deep red fluorescent cell tracker dyes and exposed to viable tachyzoites of *Toxoplasma gondii* expressing green fluorescent protein (PTG strain). (A) After the prepared inserts were turned upside down, THP-1 cells (10^5^ cells/well) were added to the basal side of the inserts. After incubation for 1 h, the inserts were placed back into the 24-well plates and incubated for another 1 h, and then transwell membranes were fixed, permeabilized, and immunostained with ZO-1 (red). The results of (B) THP-1 cells that were not infected (magenta) and (C) THP-1 cells that were infected with tachyzoites (magenta with green fluorescent dots around) were examined under the fluorescence microscope. Figures were selected as representative data from three independent experiments. Scale bar = 20 μm.

THP-1, a monocytic cell line that is derived from a patient with acute myeloid leukemia [20], were infected with freshly prepared tachyzoites. Then, infected THP-1 cells were added to the basolateral side of RPE monolayers that were prepared on the 5-μm pore-size transwell membrane, and the barrier integrity was evaluated by staining of tight junction protein (Fig 1A). Zonula occludens-1 (ZO-1) was selected among tight junction proteins because ZO-1 was more sensitive to cytokine than others in RPE cells [21]. After two hours of incubation, the transwell membranes were isolated and immunostained with anti-ZO-1 antibody. In a group incubated with uninfected THP-1 cells, ZO-1 was localized as continuous lines at the periphery of each cell, which shows its association with the membrane (Fig 1B). In a group incubated with THP-1 cells infected with *T*. *gondii*, a much granular pattern and an occasional loss of association with the membrane were observed (Fig 1C). Further analysis with additional information on the location of THP-1 cells revealed that the ZO-1 expression of RPE cells adjacent to the infected THP-1 cells (magenta with multiple green fluorescent dots around) was mainly affected, but its integrity in RPE cells that lay apart from the infected THP-1 cells was also impaired (Fig 1C).

### Infected monocytes decrease TEER

**Fig 2:**
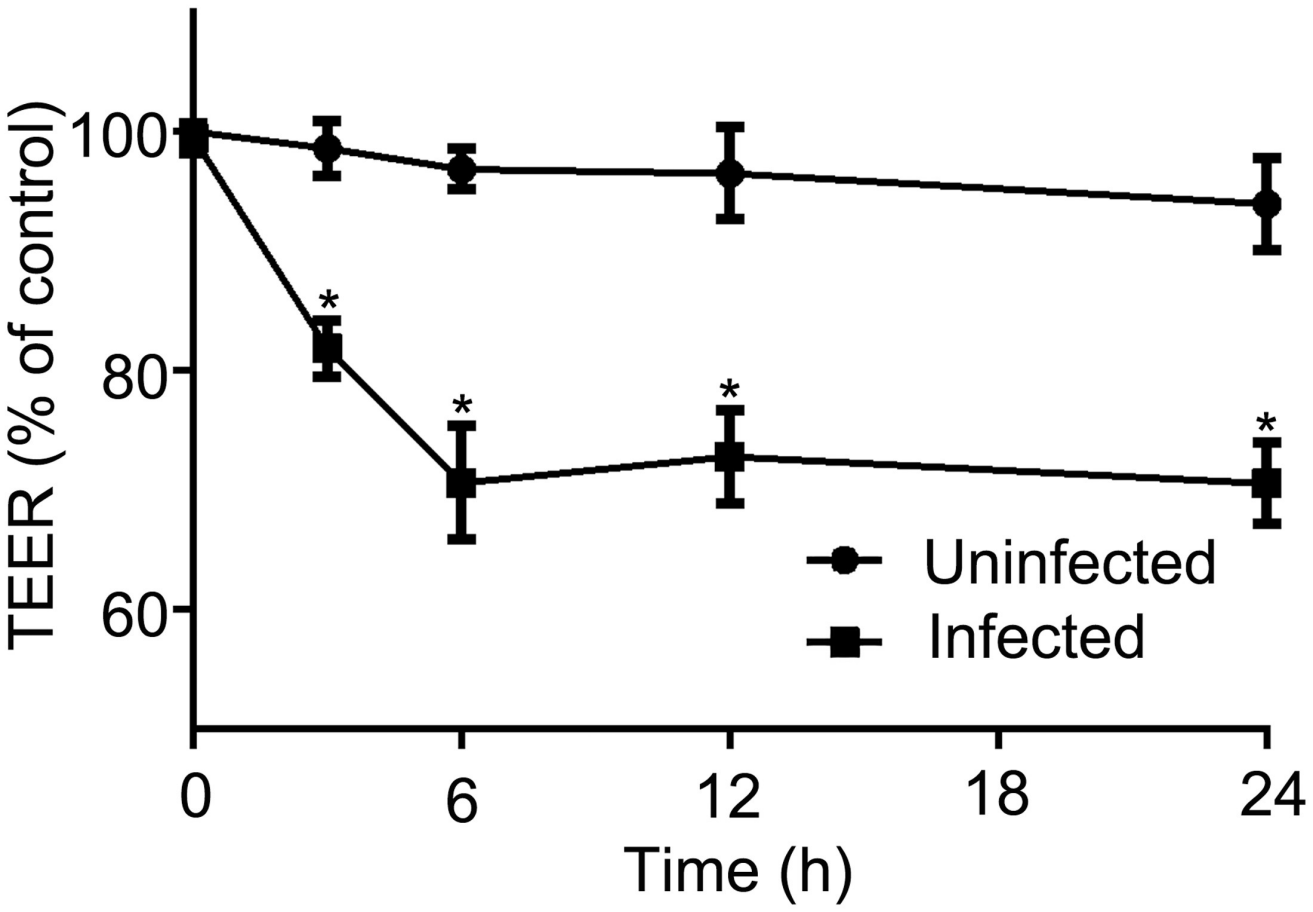
Infected monocytes decrease transepithelial electrical resistance. THP-1 cells that were not infected (Uninfected) and THP-1 cells infected with *T*. *gondii* tachyzoites (Infected) were added to RPE monolayer (10^5^ cells/well). Transepithelial electrical resistance was measured before the treatment (0 h), and 3, 6, 12 and 24 hours after the treatment. Values of uninfected group at 0 h were normalized to 100%. Data were presented as the mean ± SEM of three independent experiments. *P<0.05.

To investigate the effect of ZO-1 disruption on the permeability of outer BRB, TEER was evaluated after the addition of infected THP-1 cells. We have previously shown that TEER was decreased under the condition that ZO-1 was disrupted in RPE monolayers [22]. The mean TEER established on the RPE monolayers cultivated on the 5-μm pore-size transwell membrane was 60.6 ± 1.3 ohms▪cm^2^ when the value reached a plateau. Three and six hours after incubation with infected THP-1 cells, TEER was significantly decreased to 81.9 ± 2.4% and 70.6 ± 4.7%, respectively, while a group incubated with uninfected THP-1 cells remained unchanged (Fig 2). Further evaluation revealed minimal changes on TEER later on. Our findings of ZO-1 immunostaining and TEER measurements demonstrate infected monocytes can disrupt outer BRB.

### Conditioned media from infected monocytes can also disrupt outer BRB

**Fig 3:**
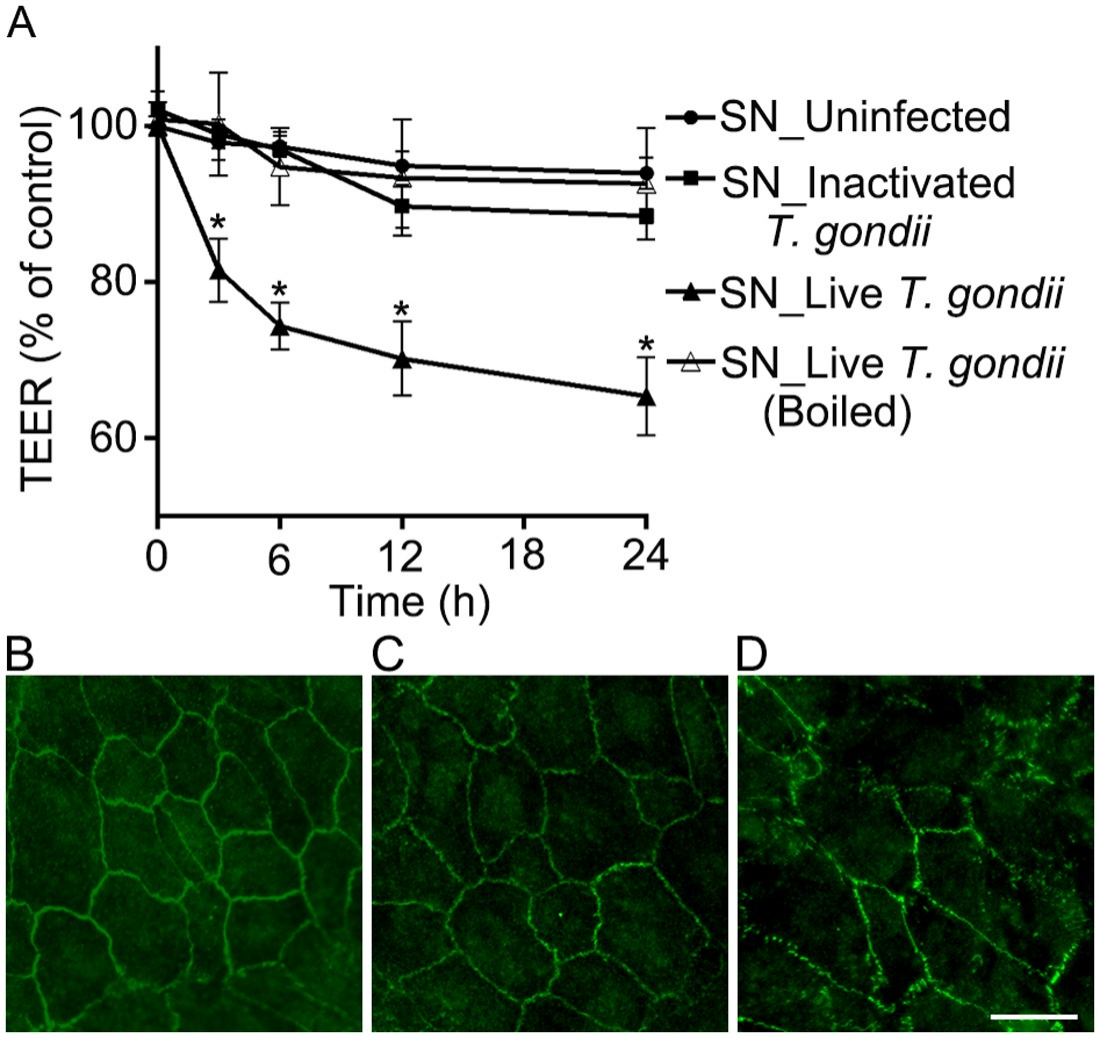
Conditioned media from infected monocytes can also disrupt outer BRB. (A) Transepithelial electrical resistance was measured before the treatment (0 h), and 3,6,12 and 24 h after the treatment of supernatants from THP-1 cells that were not infected (SN_Uninfected), THP-1 cells that were exposed to heat-inactivated tachyzoites (SN_Inactivated *T*. *gondii*) and THP-1 cells infected with live tachyzoites of *T*. *gondii* (SN_Live *T*. *gondii*). In addition, supernatants from infected THP-1 cells were boiled at 100°C for 20 min, and utilized (SN_ Live *T*. *gondii* (Boiled)). (B-D) Expression of ZO-1 was evaluated by immunocytochemical staining of ZO-1 (green) 6 h after the treatment of supernatants from THP-1 cells that were not infected (B), from THP-1 cells that were exposed to heat-inactivated tachyzoites (C) and from THP-1 cells infected with live tachyzoites of *T*. *gondii* (D). Data were presented as the mean ± SEM of five independent experiments. Figures were selected as representative data from three independent experiments. *, *P*<0.05. Scale bar = 20 μm.

Our findings of ZO-1 disruption that occurred distant from the infected monocytes and decreased TEER that infers generally increased permeability throughout the membrane, suggest the presence of factors that can work paracrinely to disrupt tight junctions without necessity for direct cell contacts. The paracrine effect of infected monocytes was evaluated by application of conditioned media isolated from infected monocytes on outer BRB. Supernatants were collected from infected THP-1 cells by centrifugation and applied to basolateral side of RPE monolayers (Fig 3). The mean TEER established on the RPE monolayers cultivated on the 0.4-μm pore-size transwell membrane was 65.8 ± 1.4 ohms▪cm^2^ when the value reached a plateau. Three and six hours after the treatment of the conditioned media from infected THP-1 cells, TEER was significantly decreased to 81.6 ± 4.0% and 74.4 ± 3.0%, respectively, while groups incubated with supernatants from uninfected THP-1 cells, supernatants from THP-1 cells exposed to heat-inactivated *T*. *gondii* tachyzoites or boiled supernatants, remained unchanged (Fig 3A). Immunostaining of ZO-1 on RPE monolayers at 6 h revealed that membrane-associated pattern of ZO-1 expression was preserved in RPE cells treated with supernatants from uninfected THP-1 cells (Fig 3B) or from THP-1 cells exposed to heat-inactivated *T*. *gondii* tachyzoites (Fig 3C), while the integrity was lost in RPE cells treated with supernatants from infected THP-1 cells (Fig 3D).

### FAK is activated by conditioned media from infected monocytes

When infected with *T*. *gondii*, human leukocytes are altered in gene expression [23]. As diverse factors secreted from infected leukocytes probably work as synergistically or antagonistically to make consequence of disrupted barrier, it is feasible to investigate common pathways mediating the disruption in RPE first.

Previous studies on animal models of ocular toxoplasmosis commonly showed migration of RPE cells in animals infected with *T*. *gondii* [24, 25]. As the migration can be triggered by disruption of RPE monolayer [24], one common pathway can probably modulate both the migration in animal models and the disruption of outer BRB in our study. In other immune privileged organs, FAK activation was found to be involved in disruption of blood-brain barrier [26] and blood-testis barrier [27]. As FAK is not only known to be closely involved in migration of cells [28], but also known to regulate tight junctions in the epithelium [29, 30], the involvement of FAK signaling was investigated.

**Fig 4:**
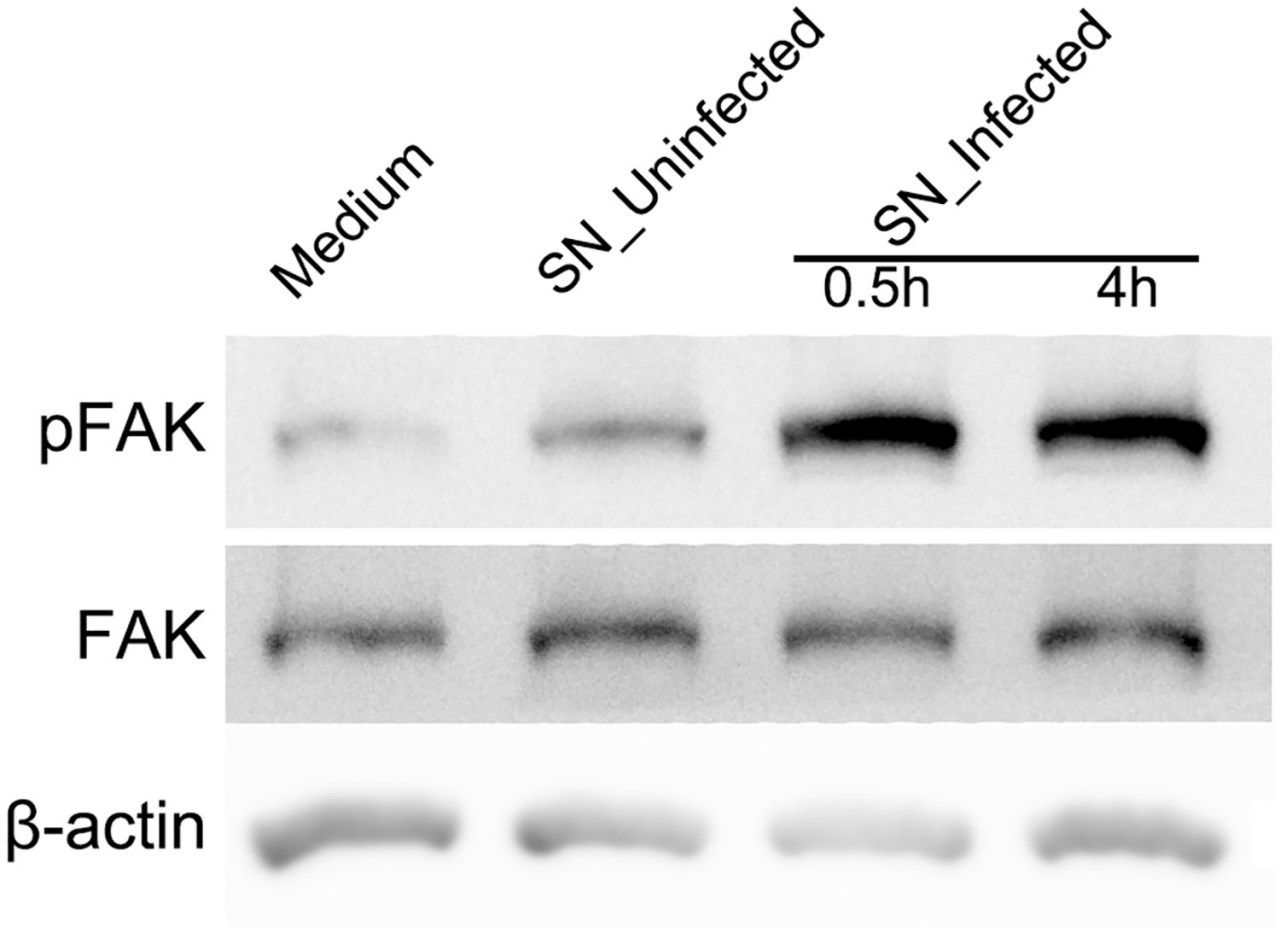
FAK is activated by conditioned media from infected monocytes. Representative Western blots of anti-pFAK, anti-FAK and anti-β-actin obtained with RPE cell lysates after treatment of conditioned media. RPE cells were incubated with standard medium, supernatants from uninfected THP-1 cells (SN_Uninfected) for 0.5 h, or supernatants from infected THP-1 cells (SN_Infected) for 0.5 and 4 h. β-actin served as loading control. Figures were selected as representative data from three independent experiments.

We first evaluated the activation of FAK by conditioned media. After the treatment of supernatants from infected THP-1 cells on RPE cells, expressions of FAK and phosphorylated FAK were determined by Western blot analysis. The ratio of phosphorylated FAK over FAK was increased 4.3- and 3.5-fold when treated with supernatants from infected THP-1 cells for 0.5 h and 4 h, respectively, compared to a group incubated with standard medium (Fig 4). In a group treated with supernatants from uninfected THP-1 cells, the ratio of phosphorylated FAK over FAK remained unchanged at 0.5 h.

### Inhibition of FAK signaling attenuates the disruption of outer BRB

**Fig 5:**
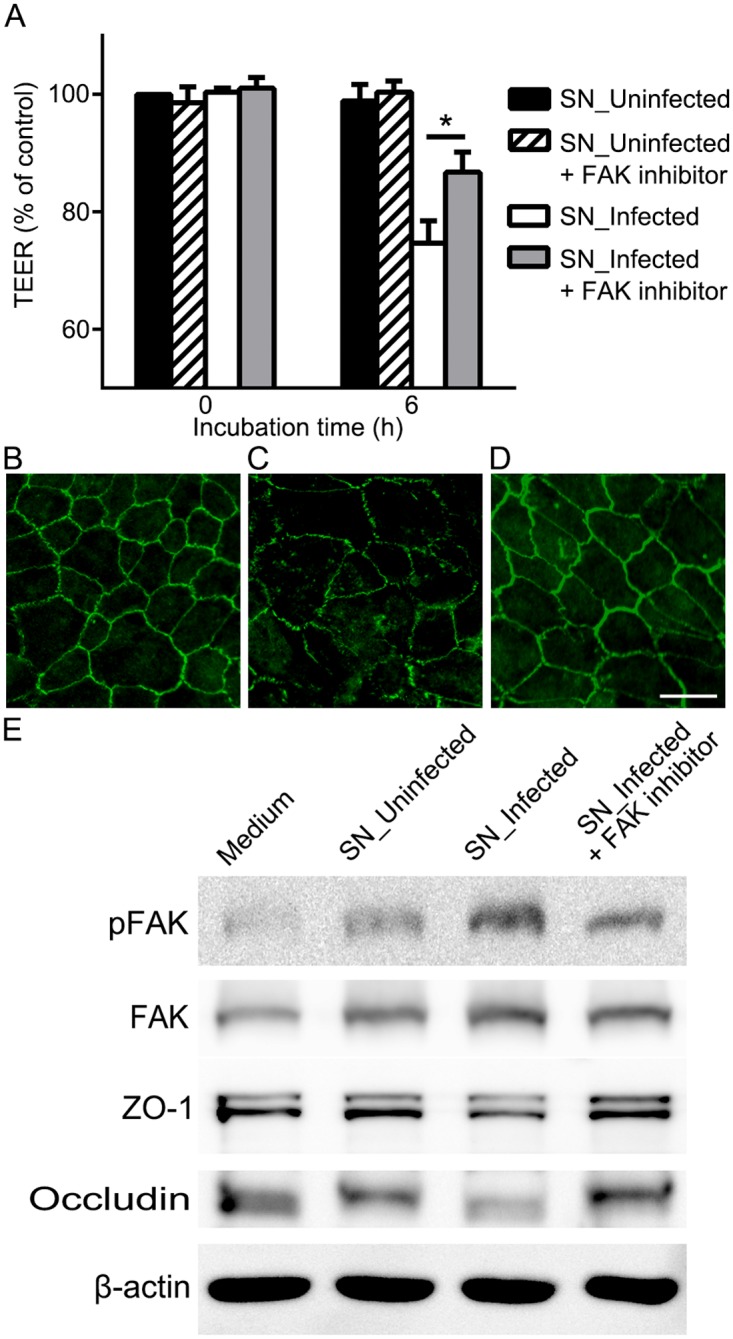
Inhibition of FAK signaling attenuates the disruption of outer BRB. (A) Transepithelial electrical resistance was measured before the treatment (0 h) and 6 h after the treatment of supernatants from THP-1 cells that were not infected (SN_Uninfected) or from THP-1 cells infected with *T*. *gondii* (SN_Infected) with or without FAK inhibitor (PF-573228, 1μM). (B-D) Expression of ZO-1 was evaluated by immunocytochemical staining of ZO-1 (green) 6 h after the treatment of supernatants from uninfected THP-1 cells with FAK inhibitor (B), supernatants from infected THP-1 cells without FAK inhibitor (C) or with FAK inhibitor (D). (E) Representative Western blots of anti-pFAK, anti-FAK, anti-ZO-1, anti-occludin and anti-β-actin obtained with RPE cell lysates after treatment of conditioned media with or without FAK inhibitor for 6 h. β-actin served as loading control. Data were presented as the mean ± SEM of five independent experiments. Figures were selected as representative data from three independent experiments. *, *P*<0.05. Scale bar = 20 μm.

Then, the effect of inhibiting FAK activation on barrier function of RPE was investigated. A selective FAK inhibitor, PF-573228, that is known to inhibit FAK phosphorylation with an IC_50_ of 30–100 nM was utilized [31]. After the FAK inhibitor was confirmed to have no effect on viability of RPE cells at a concentration of 1 μM (S1 Fig), it was added to supernatants to inhibit FAK activation. The addition of FAK inhibitor to control group did not have any adverse effect on TEER at 6 h (Fig 5A) and any other time point later on when observed until 24 h. Six hours after the treatment of supernatants from infected THP-1 cells with or without additional FAK inhibitor, TEER was higher in a group with additional FAK inhibitor (86.8 ± 3.5%) than without it (74.7 ± 3.9%) (Fig 5A). The results were in accordance with FITC-dextran permeability assay performed after 6 h incubation with corresponding supernatants. Increased FITC-Dextran permeability by supernatants from infected THP-1 cells was attenuated by addition of FAK inhibitor (S2 Fig). Immunostaining of ZO-1 on RPE monolayer fixed at 6 h revealed corresponding results with TEER measurement. The membrane-associated pattern of ZO-1 expression was preserved in RPE cells treated with addition of FAK inhibitor to control group (Fig 5B). The loss of integrity in RPE cells by supernatants from infected THP-1 cells (Fig 5C) was attenuated by addition of FAK inhibitor (Fig 5D).

The effect of FAK inhibitor was further evaluated with Western blot analysis of phosphorylated FAK and tight junction proteins. The ratio of phosphorylated FAK over FAK was increased 2.6-fold at 6 h after the treatment with supernatants from infected THP-1 cells, but addition of FAK inhibitor significantly attenuated the ratio increase to half (Fig 5E). Further evaluation with the corresponding samples demonstrated that decreased expressions of tight junction proteins, ZO-1 and occludin, after the treatment of supernatants from infected THP-1 cells were restored by addition of FAK inhibitor (Fig 5E).

Previous studies on the association between FAK and tight junction proteins demonstrated that increased association of FAK with occludin destabilize occludin/ZO-1 protein-protein interaction and disrupt blood-testis barrier [27]. Inhibition of FAK activation was sufficient to prevent ZO-1 disruption in blood-brain barrier [26]. Here, we demonstrated the activation of FAK signaling during the disruption and protective effect of inhibiting FAK activation on barrier integrity in outer BRB.

### Blockade of CXCL8 can partly rescue the disruption of outer BRB

To investigate factors in supernatants mediating the disruption, secretory factors from infected leukocytes were evaluated by analyzing Gene Expression Omnibus (GEO) database (NCBI GEO accession no. GSE360) regarding gene expression of monocyte-derived macrophages infected with *T*. *gondii*. The expression values were compared to the untreated macrophages, and genes were sorted in ascending order of adjusted p-value calculated by GEO2R provided by GEO. Chemokine (C-X-C motif) ligand 8 (CXCL8; ID: 35372_r_at) had lowest adjusted p-value among genes producing secretory factors. The expression value increased more than nine-fold after the infection while it increased less than two-fold after exposure to extracellular parasites, *Brugia malayi*. Furthermore, CXCL8 is well known to induce FAK phosphorylation via CXCL8 receptors CXCR1 and CXCR2 [32] and CXCL8 receptors are known to be expressed in RPE [33]. Thus, the role of CXCL8 in the disruption of outer BRB by supernatants from infected THP-1 cells was investigated.

**Fig 6:**
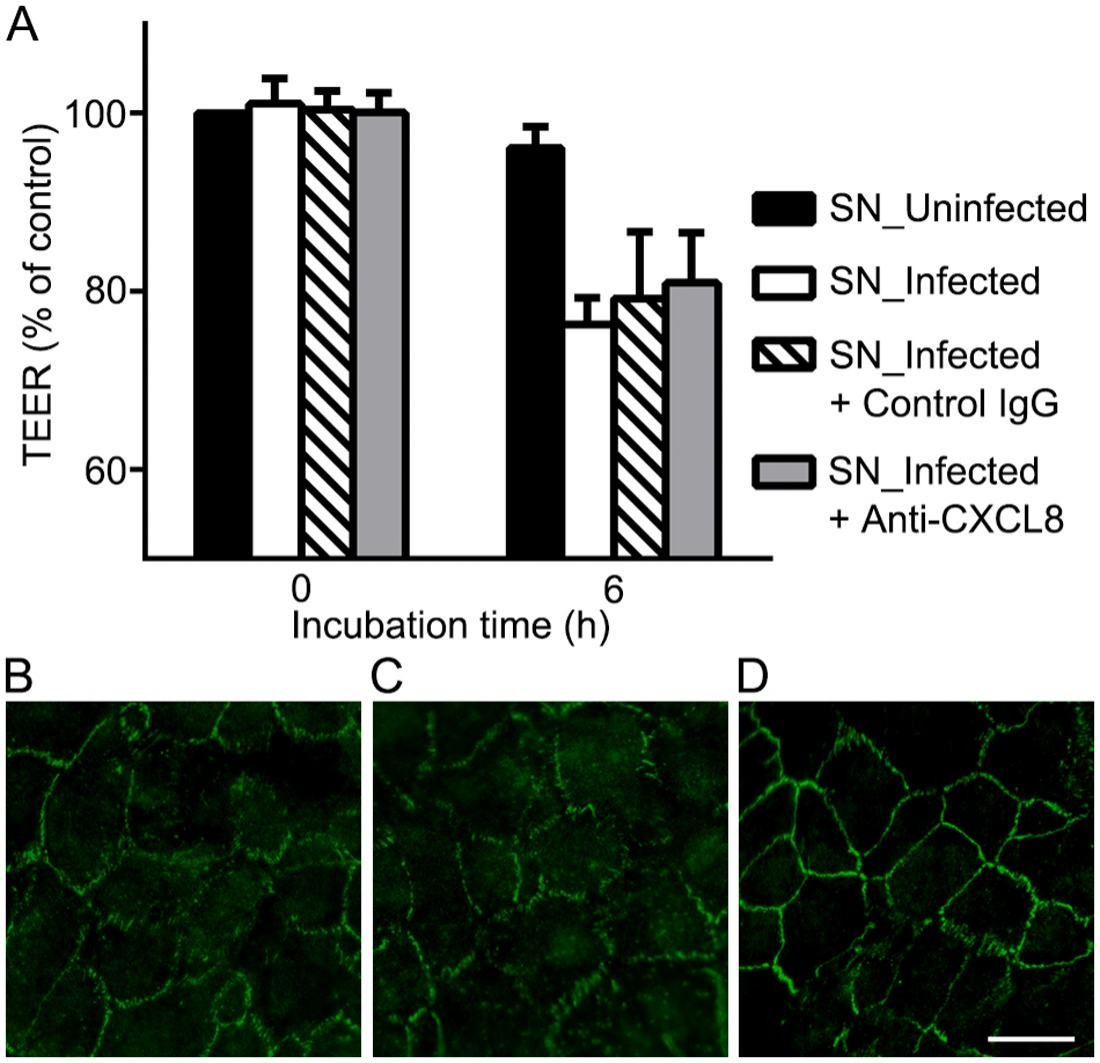
Blockade of CXCL8 can partly rescue disruption of outer BRB. (A) Transepithelial electrical resistance was measured before the treatment (0 h) and 6 h after the treatment of supernatants from uninfected THP-1 (SN_Uninfected) or from infected THP-1 cells (SN_Infected) with a control IgG or with a neutralizing antibody against CXCL8 (Anti-CXCL8, 1 μg/mL). (B-D) Expression of ZO-1 was evaluated by immunocytochemical staining of ZO-1 (green) 6 h after the treatment of supernatants from infected THP-1 cells (B), and additional treatment with control IgG (C) or neutralizing antibodies against CXCL8 (D). Data were presented as the mean ± SEM of three independent experiments. Figures were selected as representative data from three independent experiments. Scale bar = 20 μm.

The concentration of CXCL8 in the supernatants from infected THP-1 cells was first evaluated by ELISA. Twenty-four hours after infection with *T*. *gondii*, the concentration of CXCL8 was increased to 18.97 ± 2.1 ng/mL while conditioned media from uninfected THP-1 remained undetectable. Then, a neutralizing antibody against CXCL8 was prepared to block the action of CXCL8. The monoclonal antibody had 50% neutralization dose at 0.08–0.4 μg/mL against 20 ng/mL of recombinant human CXCL8. When 1 μg/mL of the neutralizing antibody was added to supernatants from infected THP-1 cells, RPE monolayers showed slightly higher TEER but failed to show significance at 6 h (Fig 6A). Higher dosage up to 5 μg/mL failed to show additional effect on TEER at any time point. However, immunocytochemistry of ZO-1 demonstrated focal restoration of disrupted tight junction protein compared to the ZO-1 distribution after the treatment of supernatants from infected THP-1 cells with or without control IgG (Fig 6B–6D). Focal protective effects of CXCL8 blockade fail to attenuate TEER changes that measure the electrical resistance across the whole membrane. Furthermore, when relative amounts of cytokine levels in the supernatants were analyzed by cytokine array kit, other cytokines such as CCL2, CCL5, IL-1β, and IL-1ra were also increased, although to a lesser degree than CXCL8 (S3 Fig). We can suggest that although CXCL8 is not the dominant factor mediating disruption of RPE monolayers by conditioned media from infected monocytes, it can partly mediate the disruption.

The outer BRB is formed by RPE cells and fenestrated choriocapillaries while inner BRB is formed by non-fenestrated endothelium [34]. The outer BRB is considered as a site for retinal immunosurveillance where immune cells migrate to remove dead photoreceptors and cell debris in the injured retina [35]. Although there is a possibility that outer BRB during immunosurveillance was transiently disrupted as it was shown during leukocyte transmigration in retinal venules [21], it is interesting to note that obvious breakdown of the BRB was not observed during the transmigration [35]. In contrast, tight junction proteins were significantly disordered during experimental autoimmune uveoretinitis [21], which corresponds to this study that showed monocytes infected with intracellular parasites disrupted outer BRB. The discrepancy in the aspect of tight junction between immunosurveillance and our study probably depends on the status of leukocytes whether they are in the resting state or inflamed state.

The status of RPE is also important in the interactions with immune cells. CXCL8 is secreted constitutively from RPE at concentration less than 1 ng/mL on both apical and basal sides [36]. When RPE is stimulated from the apical side by mixture of IL-1β, TNF-α and IFN-γ, CXCL8 secretion from apical side is increased to 111 ng/mL while stimulation from the basal side induces only 22 ng/mL from the apical side [36]. CXCL8 secretion from the basal side remains less than 5 ng/mL in both stimulation [36]. This kind of polarized secretion can work as protective mechanism against infected cells to infiltrate RPE monolayer. When inflammation is active on the apical side where retina lies, the gradient of CXCL8 helps leukocytes infiltrate and fight against the source of inflammation. However, when inflammation is active on the basal side where choroid lies, immune cells are less likely to migrate across the RPE. In case of *T*. *gondii* infection, clinically observed punctate outer retinal toxoplasmosis [6] may correspond to the stimulation from basal side and manifest as focal lesion limited to RPE affected by chemokine from immune cells that could not infiltrate. Another manifestation may correspond to the stimulation from apical side due to the neuroretinitis and manifest as necrotizing retinochoroiditis [6] that involves whole retinal layer infiltrated with immune cells.

In this study, we demonstrated outer BRB disruption by *T*. *gondii*-infected monocytes. Supernatants alone separated from infected monocytes could also decrease TEER and disrupt tight junction proteins. During the disruption, FAK signaling was paracrinely activated. FAK inhibitor could attenuate the disruption by suppressing FAK activation. Blockade of CXCL8, a FAK activating factor in the supernatants, could partly rescue the disruption. Selectively targeting FAK activation in RPE cells can be a new strategy to reduce complications of ocular toxoplasmosis.

## Supporting information

S1 Fig Cell viability of ARPE-19 cells after incubation with FAK inhibitor (PF-573228) at various concentrations for 48h. (TIF)

S2 Fig FITC-Dextran permeability assay in ARPE-19 cells after 6 h incubation with supernatants. (TIF)

S3 Fig Human cytokine array analysis of supernatants from infected THP-1 cells. (TIF)

S1 File Complete data set for Figs 2, 3, 5 and 6, and S1 and S2 Figs. (XLSX)

## Abbreviations

BRB: Blood-retinal barrier
FAK: focal adhesion kinase
HFF: human foreskin fibroblast
*T. gondii*: *Toxoplasma gondii*
TEER: Transepithelial electrical resistance
