# Supplementary figures and images for: Disruption of outer blood-retinal barrier by Toxoplasma gondii-infected monocytes is mediated by paracrinely activated FAK signaling
Source: PLoS One. 2017 Apr 13;12(4):e0175159. doi: 10.1371/journal.pone.0175159 (PMC5390985; doi:10.1371/journal.pone.0175159)

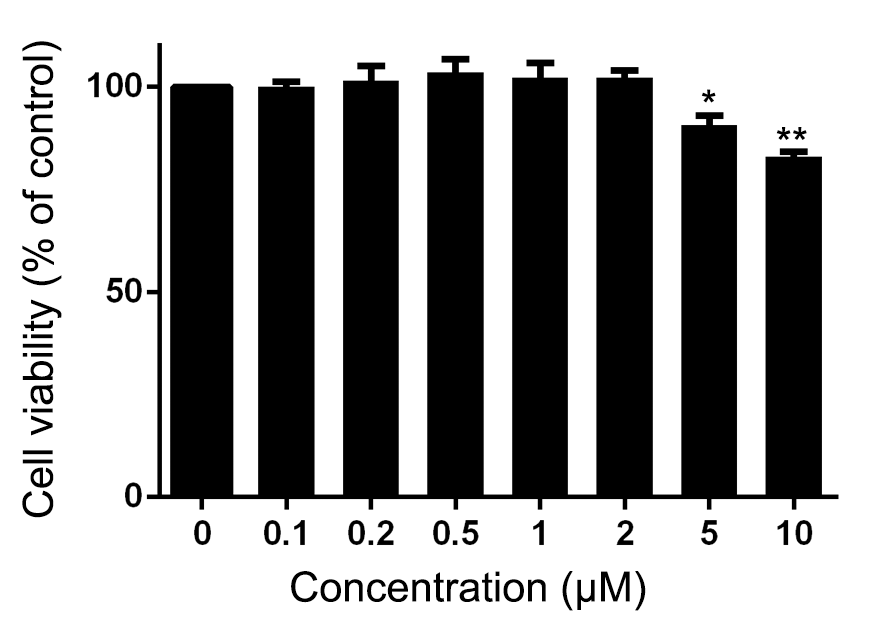

Supplement: S1 Fig — (TIF) [file pone.0175159.s001.tif]

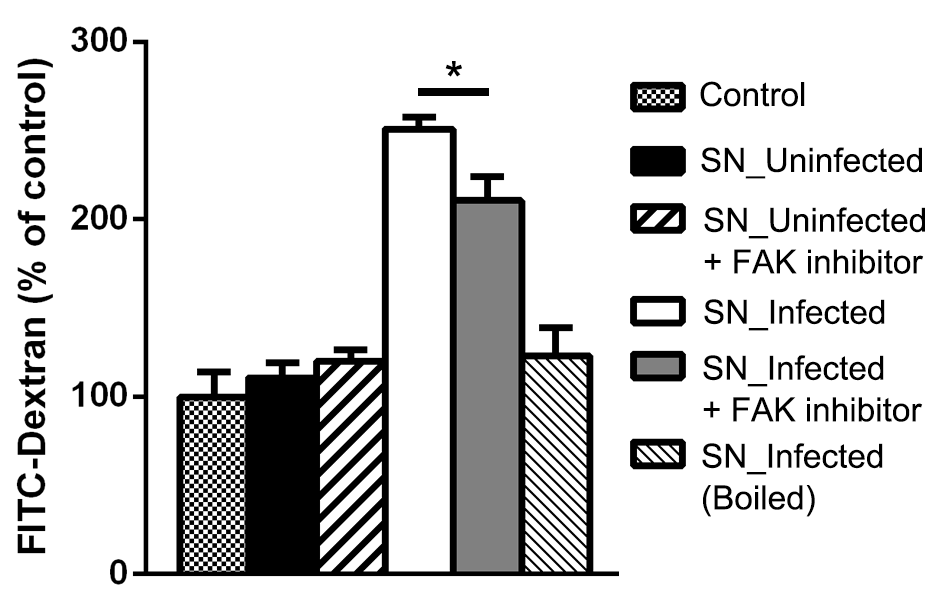

Supplement: S2 Fig — (TIF) [file pone.0175159.s002.tif]

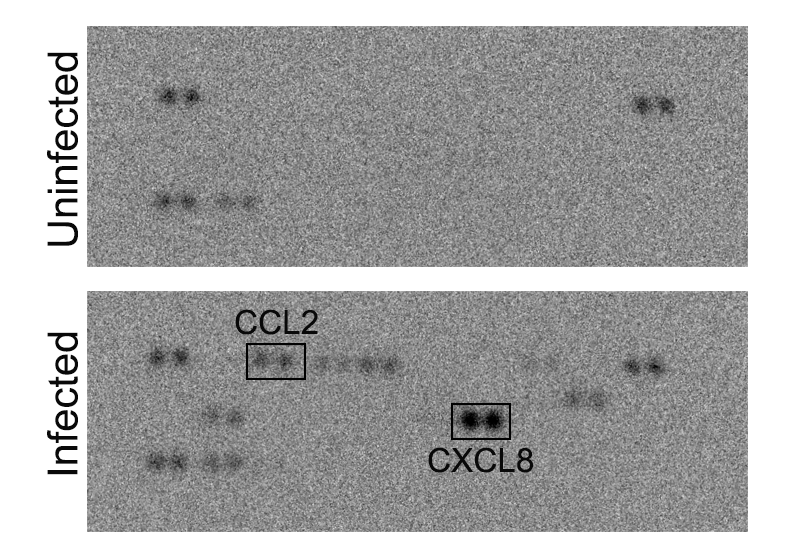

Supplement: S3 Fig — (TIF) [file pone.0175159.s003.tif]
